# Aortic flow is abnormal in HFpEF

**DOI:** 10.12688/wellcomeopenres.20192.1

**Published:** 2023-12-15

**Authors:** Zia Mehmood, Hosamadin Assadi, Rui Li, Bahman Kasmai, Gareth Matthews, Ciaran Grafton-Clarke, Aureo Sanz-Cepero, Xiaodan Zhao, Liang Zhong, Nay Aung, Kristian Skinner, Charaka Hadinnapola, Peter Swoboda, Andrew J. Swift, Vassilios S Vassiliou, Christopher Miller, Rob J. van der Geest, Steffen Peterson, Pankaj Garg

**Affiliations:** 1 Department of Cardiology, Norfolk and Norwich University Hospitals NHS Foundation Trust, Norwich, Norfolk, NR4 7UY, UK; 2 Department of Cardiovascular and Metabolic Health, Norwich Medical School, University of East Anglia, Norwich, Norfolk, NR4 7TJ, UK; 3 National Heart Research Institute, National Heart Centre Singapore, Singapore, 169609, Singapore; 4 Cardiovascular Sciences Academic Clinical Program & Cardiovascular Metabolic Disorder Program, Duke National University of Singapore Medical School, Singapore, 169857, Singapore; 5 Department of Biomedical Engineering, National University of Singapore, Singapore, 117583, Singapore; 6 William Harvey Research Institute, NIHR Barts Biomedical Research Centre, Queen Mary University of London, London, EC1M 6BQ, UK; 7 Barts Heart Centre, St Bartholomew’s Hospital, Barts Health NHS Trust, London, EC1A 7BS, UK; 8 Division of Biomedical Imaging, Leeds Institute of Cardiovascular and Metabolic Medicine, University of Leeds, Leeds, LS2 9JT, UK; 9 Department of Infection, Immunity & Cardiovascular Disease, The University of Sheffield, Sheffield, UK; 10 Division of Cardiovascular Sciences, School of Medical Sciences, Faculty of Biology, Medicine and Health, Manchester Academic Health Science Centre, The University of Manchester, Manchester, UK; 11 Department of Radiology, Division of Image Processing, Leiden University Medical Center, Leiden, 2300 RC, The Netherlands

**Keywords:** Magnetic Resonance Imaging, Aortic Flow, Haemodynamics, HFpEF, Cardiac Output

## Abstract

**Aims:**

Turbulent aortic flow makes the cardiovascular system less effective. It remains unknown if patients with heart failure with preserved ejection fraction (HFpEF) have disturbed aortic flow. This study sought to investigate advanced markers of aortic flow disturbances in HFpEF.

**Methods:**

This case-controlled observational study used two-dimensional phase-contrast cardiovascular magnetic resonance data at an orthogonal plane just above the sino-tubular junction. We recruited 10 young healthy controls (HCs), 10 old HCs and 23 patients with HFpEF. We analysed average systolic aortic flow displacement (FDsavg), systolic flow reversal ratio (sFRR) and pulse wave velocity (PWV). In a sub-group analysis, we compared old HCs versus age-gender-matched HFpEF (N=10).

**Results:**

Differences were significant in mean age (P<0.001) among young HCs (22.9±3.5 years), old HCs (60.5±10.2 years) and HFpEF patients (73.7±9.7 years). FDsavg, sFRR and PWV varied significantly (P<0.001) in young HCs (8±4%, 2±2%, 4±2m/s), old HCs (16±5%, 7±6%, 11±8m/s), and HFpEF patients (23±10%, 11±10%, 8±3). No significant PWV differences existed between old HCs and HFpEF.HFpEF had significantly higher FDsavg versus old HCs (23±10% vs 16±5%, P<0.001). A FDsavg > 17.7% achieved 74% sensitivity, 70% specificity for differentiating them. sFRR was notably higher in HFpEF (11±10% vs 7±6%, P<0.001). A sFRR > 7.3% yielded 78% sensitivity, 70% specificity in differentiating these groups. In sub-group analysis, FDsavg remained distinctly elevated in HFpEF (22.4±9.7% vs 16±4.9%, P=0.029). FDsavg of >16% showed 100% sensitivity and 70% specificity (P=0.01). Similarly, sFRR remained significantly higher in HFpEF (11.3±9.5% vs 6.6±6.4%, P=0.007). A sFRR of >7.2% showed 100% sensitivity and 60% specificity (P<0.001).

**Conclusion:**

Aortic flow haemodynamics namely FDs _avg_ and sFRR are significantly affected in ageing and HFpEF patients.

## Abbreviations

2D                two-dimensional

4D                four-dimensional

CMR           cardiovascular magnetic resonance imaging

FD                flow displacement

FDs _avg_          average systolic flow displacement

HCs               healthy controls

HFpEF           heart failure with preserved ejection fraction

HFrEF           heart failure with reduced ejection fraction

LVFP           left ventricular filling pressure

LVEF           left ventricular ejection fraction

PC                phase contrast

PWV              pulse wave velocity

sFRR            systolic flow reversal ratio

RA                rotational angle

## Introduction

Heart failure with preserved ejection fraction (HFpEF) is a heterogeneous clinical syndrome in which patients have signs and symptoms of heart failure. The diagnosis of HFpEF has a significant negative impact on quality of life and a prognostic outcome comparable to those with heart failure with reduced ejection fraction (HFrEF) ^1, 2^. The hallmark pathophysiology of HFpEF is high left ventricular (LV) filling pressure (LVFP) despite normal or near normal LV ejection fraction (LVEF; ≥50 per cent) ^3^. The epidemiology of heart failure with preserved ejection fraction (HFpEF) is constantly evolving as the condition continues to be a significant global health concern, with a prevalence of 2-3% in the general population and up to 50% in the elderly population ^2, 4^. There is a higher prevalence of hypertension, atrial fibrillation (AF), coronary artery disease (CAD), dyslipidemia, obesity, anaemia, diabetes mellitus, chronic kidney disease, and sleep-disordered breathing in these patients ^2, 5, 6^.

The aorta is subject to unique and complex flow dynamics, characterised by high flow rates, extreme pressure variations, and intricate flow patterns in both physiological and pathological states ^7^. The relationship between arterial stiffness and LV diastolic function is well established ^8– 10^. Numerous studies have demonstrated and established quantitative aortic flow parameters such as flow displacement (FD) and flow reversal ratio (FRR) in pathological states such as aortopathy and aortic valve disease ^7, 11– 13^. Elevated systolic flow displacement (FDs _avg_), a marker of aortic flow eccentricity, causes turbulence within the ascending aorta. Any increase in FD leads to a rise in energy dissipation, which in turn reduces the efficiency of the cardiovascular system.

Additionally, any increase in systolic flow reversal ratio (sFRR) in the ascending aorta causes a loss of forward flow which is detrimental to the aortic conduit function, directly resulting in reduced peripheral perfusion and tissue oxygenation ^7^. This intricate interplay between aortic haemodynamics and left ventricular function, referred to as ventricular-arterial coupling ^14– 17^, remains insufficiently researched. Even though specific aetiological factors of HFpEF, for example, hypertension or diabetes, have been associated with aortic stiffness, it remains unknown if patients with HFpEF have aortic flow abnormalities described above, which can result in heightened cardiac workload, decreased cardiac output and compromised distal perfusion subsequently causing shortness of breath.

We hypothesise that patients with HFpEF have signs of abnormal aortic flow which compromises the aortic conduit function and results in more energy expenditure, making the cardiovascular system less efficient. Hence, the main objective of this study was to investigate aortic flow haemodynamics utilising four-dimensional (4D) flow cardiovascular magnetic resonance (CMR) imaging in patients with HFpEF and healthy controls.

## Methods

### Patient and Public Involvement

The engagement of patients and the public was initiated at the project's inception through Norfolk and Suffolk Primary and Community Care Research Office ( https://nspccro.nihr.ac.uk/working-with-us/public-patient-and-carer-voice-in-research). The PPI panel helped to make the study protocol patient friendly. PPI group provided insight into design of patient information sheet for the study. PPI group were in agreement that the research will produce open access research papers available to all to read.

### Study cohort

We identified patients from the PREFER-CMR registry **(ClinicalTrials.gov: NCT05114785**). We enrolled 20 subjects into the healthy control (HC) group. The main inclusion criteria for the HCs were: > 18 years of age (<30 years for young HCs and, >50 years for old HCs) and no prior history of cardiovascular disease. We enrolled 23 patients with HFpEF. The main inclusion criteria for patients were over 18 years of age and a confirmed diagnosis of HFpEF by CMR. The exclusion criteria were limited to any CMR contraindication.

### Ethics approval and consent to participate

This study was conducted according to the principles outlined in the Declaration of Helsinki - Version 2013. The collection and management of data were approved by the National Research Ethics Service in the United Kingdom (21/NE/0149). A pragmatic opt-out informed consent was obtained from all subjects included in the study ^18^.

### Cardiovascular Magnetic Resonance protocol

CMR study was performed on a 1.5 Tesla Magnetom Sola Siemens system with a superconducting magnet (Siemens Healthineers AG, Erlangen, Germany). All patients were examined in the supine position, headfirst, using a respiratory sensor and electrocardiogram gating. Additionally, the scanner was equipped with a Biometric body with 18 coils.

The CMR protocol included baseline survey images and cines, gadolinium enhancement imaging, and 4D flow acquisition methods previously described by our group ^17, 19– 24^. If 4D flow was not available, two-dimensional phase contrast acquisition was done at an orthogonal plane just above the sino-tubular junction.

For standard cines, we acquired 30 phases throughout the cardiac cycle. Other cine acquisition parameters include TR: 2.71, TE: 1.13, field of view (FOV): 360 × 289.3mm2 with Phase FOV – 80.4%, number of signal averages (NSA): 1, matrix: 224 × 180 [phase], bandwidth: 167.4 kHz, [930Hz/Px], flip angle: 80, slice thickness: 8 mm and Grappa acceleration with a factor of 2.

For 4D flow acquisition, the initial VENC setting was 150–200 cm/s for all HCs and HFpEF cases. For 4D flow, we acquired 30 phases throughout the cardiac cycle to keep the data consistent with cines. The acquired temporal resolution was 40 ms. Other 4D flow acquisitions parameters include TR: 4.98, TE: 2.71, field of view (FOV): 200 × 256.3 mm2, number of signal averages (NSA): 1, acquired voxel size = 3 × 3 × 3 mm3, bandwidth: 31.616 kHz, [494Hz/Px], flip angle: 5, and Grappa acceleration in the phase-encoding direction with a factor of 2 and slice direction of 1. The electrocardiogram was retrospectively gated with free breathing to avoid diastolic temporal blurring.

### CMR analysis

**Figure 1.**
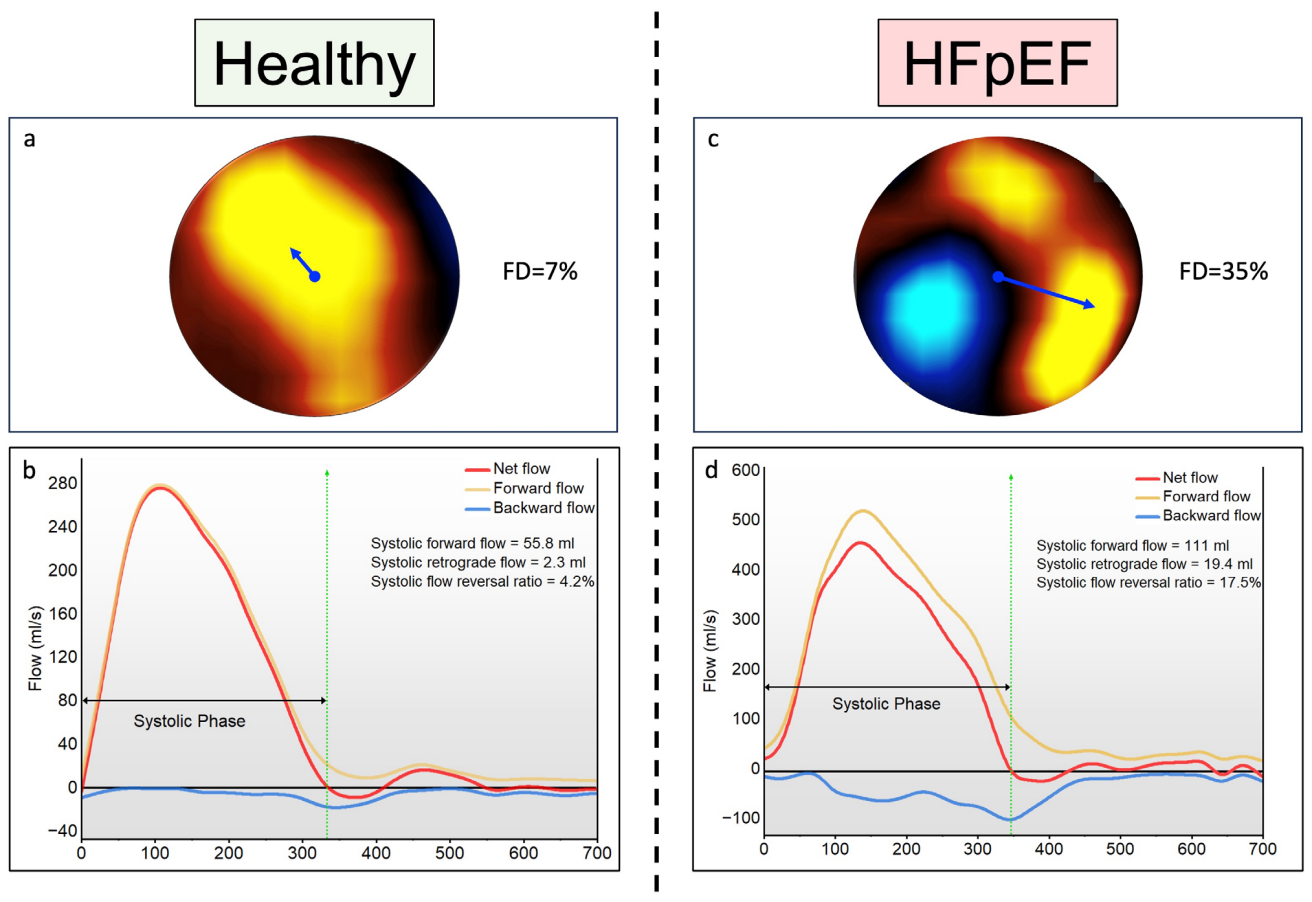
Central illustration of case examples from the study cohort. **A** and **B** – Flow mapping demonstrates normal predominantly laminar flow at peak systole in healthy individual with minimal flow reversal during systolic phases. **C** and **D** – Flow mapping a HFpEF patient demonstrating significant shift in flow displacement at peak systole. There is clear flow reversal observed (blue zone) at peak systole which is affecting aortic forward flow. There is quantified by FRR which is 17.5% during systole and can be noted on backward flow mapping (blue line) on the aortic flow curves.

All image analyses were post-processed with the in-house developed MASS research software (MASS; Version 2022-EXP, Leiden University Medical Center, Leiden, The Netherlands). A static reformatted plane was planned through the ascending aorta at the mid-main pulmonary artery level to generate a through-plane velocity encoded two-dimensional (2D) phase contrast (PC) data using 4D flow CMR data. This plane was treated as a two-dimensional phase contrast plane. Ascending aortic helical flow was defined as the flow swirling around the aortic centre line. Ascending aorta vortex flow was defined as any flow rotating on the long axis of the aorta near the inner curvature of the aortic root ^25^ ( Figure 1). The following parameters we automatically derived based on the aortic contours:

- Aortic Forward Flow: This refers to the stroke volume during a cardiac cycle.
- Aortic maximum and minimum area are the largest and smallest cross-sectional area (respectively) computed in the ascending aorta during a cardiac cycle.
- Flow displacement systolic average (FDsavg): This is the distance between the vessel’s central point and the centre-of-velocity of the forward flow, normalised to the vessel size during systole. It is presented as a percentage. The centre-of-velocity of the forward flow is computed as the mean location of pixels weighted by the velocity values within a defined aortic contour on a 2D Phase Contrast (PC) image.
- Rotational Angle signifies the angle formed by the line connecting the entre of forward flow velocity and the radius pointing at 12 o’clock position within the ascending aorta cross-section on every 2D PC image during cardiac cycle. RA was established at zero when the vector pointed at 12 o’clock and progressed clockwise to 180 degrees when pointing backward (6 o’clock position). This is sensitive to errors in negligible flow displacement as the position of the centre-of-velocity and vessel are at a close proximity. Therefore, a slight modification of the aortic contour can displace the location of the centre-of-velocity flow and significantly alter RA. To circumvent this effect, we chose a FD=12% threshold based on our bench testing.
- Systolic Forward and Backward Flows: These are obtained using per-pixel information. All positive velocities within the region of interest during systole were used to derive systolic forward flow. In contrast, all negative velocities within the same region were used to derive systolic negative flow.
- Systolic Flow Reversal Ratio Flow curves generated for each cardiac cycle. sFRR= systolic retrograde flow/systolic forward flow x 100%.
- Pulse Wave Velocity is the ratio of distance and transit time between ascending to descending aorta. A 3D image is created from parallel scout images of ascending, arch and descending aorta. 2D PC aortic flow plane matched to 3D images noting exact velocity measure points. Arch length is traced between these points. Transit time, calculated as the time difference between the points where the ascending and descending aorta flow waveforms reached half maximum of their peak velocities.

### Statistical analysis

Data analyses were performed using MedCalc® Statistical Software, version 20.215 (MedCalc Software Ltd., Ostend, Belgium) and OriginPro, version 2023 (OriginLab Corporation, Northampton, MA, USA). Continuous variables are presented as the median along with the interquartile range (IQR). We treated data as non-parametric. To compare variables between the two groups, we employed the Mann-Whitney U test. For testing across three groups, we utilised the Kruskal-Wallis one-way ANOVA and followed it with a post-hoc analysis using the Conover method. To explore the independent association of all CMR indices, we used a partial correlation factor generated via the multiple regression method using all other CMR variables as covariates. To evaluate the ability of these blood flow parameters to distinguish HFpEF, we conducted Receiver Operator Characteristic (ROC) analysis and used Youden Index to determine cut-off values. We deemed statistical significance at a threshold of P<0.05.

### Study size

We used healthy young versus old systolic flow displacement data to derive the possible sample size to differentiate healthy old versus HFpEF cohorts. Factoring a mean difference of 8% and standard deviations of 4 and 5, to achieve a alpha of 0.05, we need to recruit at least 11 HFpEF patients and 6 elderly patients with a total sample size of 17 to achieve a power of beta=0.20. To further improve the diagnostic range, we planned to recruit >20 HFpEF cases and at least 10 elderly healthy subjects.

## Results

### Study population

Patient characteristics are summarised in Table 1. The study comprised 43 cases with comparable body surface area, of which females accounted for 44%. These included ten individuals from the young HC group (females: 10%), another ten from the old HC group (females: 60%) and twenty-three HFpEF patients (females: 52%). The mean age was found to be significantly different in all three groups- younger HCs (22.9 ± 3.5 years) vs older HCs (60.5±10.2 years; P<0.001) vs HFpEF cohorts (73.7±9.7 years; P<0.001). The average age of HFpEF patients was found to be significantly older than that of old HCs (P<0.001). Among the HFpEF patients, 16 (70%) had dyslipidemia, 12 (52%) had atrial fibrillation, 11 (48%) had hypertension, 8 (35%) had coronary artery disease, and 5 (22%) were diabetics.

**Table 1.** Study demographics.

	Young HCs ^10^	Old HCs ^10^	HFpEF ^23^	P
Demographics				
Age, years	22.9 ± 3.5	60.5 ± 10.2 *	73.7 ± 9.7 ^#^	< 0.001
Female gender	1 (10)	6 (60)	12 (52)	0.076
BSA, m ^2^	1.9 ± 0.30	1.8 ± 0.20	1.93 ± 0.27	0.409
LV function				
LV mass, g/m ^2^	116 ± 19	77.6 ± 14 *	117 ± 61 ^#^	0.005
LVEDV, ml/m ^2^	194 ± 43	146 ± 40 *	146 ± 53	0.006
LVESV, ml/m ^2^	82 ± 29	55 ± 16 *	62 ± 26	0.010
LVSV, ml/m ^2^	104 ± 35	84 ± 22 *	77 ± 34	0.014
LV ejection fraction, %	56 ± 6	60 ± 9	56 ± 7	0.438
RV function				
RVEDV, ml/m ^2^	207 ± 49	144 ± 34 *	144 ± 59	0.010
RVESV, ml/m ^2^	93 ± 30	56 ± 23 *	58 ± 29	0.008
RVSV, ml/m ^2^	107 ± 39	80 ± 13	74 ± 38	0.066
RV ejection fraction, %	54 ± 5	58 ± 7	56 ± 14	0.276

Data were represented as median ± IQR (%). *BSA* body surface area, *EDV* end-diastolic volume, *ESV* end-systolic volume, *HCs* healthy cohorts, *HFpEF* heart failure with preserved ejection fraction, *LV* left ventricle, *RV* right ventricle, *SV* stroke volume. *P<0.05 compared young HCs versus old HCs; ^#^P<0.05 compared old HCs versus HFpEF.

Both the old controls and patients with HFpEF exhibited a notable decrease in aortic forward flow in comparison to young controls (P=0.02) independent of gender and despite no significant variance in the left ventricular ejection fraction ( Table 2). There was a significant rise in FDs _avg_ in old controls and HFpEF in contrast to young controls with marked differences observed between these two groups as well (16±5% vs 23±10%, P<0.001). Likewise, the sFRR significantly differed in all three groups (P<0.001). Importantly, individuals with HFpEF exhibited significantly higher sFRR compared to old controls (11±10% vs 7±6%, P<0.001). These differences in FDs _avg_ and sFRR in these two groups persisted regardless of the gender. Although the mean values of pulse wave velocity (PWV) in both old HCs and HFpEF were significantly higher than in controls, no significant differences were observed between these groups.

**Table 2.** Aortic flow indices trend across the three groups.

	Young HCs ^10^	Old HCs ^10^	HFpEF ^23^	P
Aortic flow parameters				
AO Forward Flow, ml	97±35	70±18 *	70±19	0.02
AO Forward Flow indexed, ml/m ^2^	48±14	38±15 *	36±10	0.01
AO Backward Flow, ml	2±1	1±4	2±3	0.86
AO Backward Flow indexed, ml	1±1	1±2	1±1	0.93
AO Max Area, mm ^2^	7±2	9±3	10±5	0.01
AO Min Area, mm ^2^	5±1	7±2 *	8±4	<0.001
Flow Displacement systolic average, %	8±4	16±5 *	23±10 ^#^	<0.001
Rotational Angle, °	0±0	-3±16	16±48 ^#^	0.04
Systolic Forward Flow, ml	90±37	66±18	77±27	0.15
Systolic Retrograde Flow, ml	2±1	4±4 *	7±6 ^#^	<0.001
Systolic Flow Reversal Ratio, %	2±2	7±6 *	11±10 ^#^	<0.001
Pulse Wave Velocity, m/s	4±2	11±8 *	8±3	0.03

Data were represented as median ± IQR (%). *AO* aorta, *HCs* healthy cohorts, *HFpEF* heart failure with preserved ejection fraction. *P<0.05 compared young HCs versus old HCs; ^#^P<0.05 compared old HCs versus HFpEF.

### Association of aortic flow indices with age - young HCs vs old HCs

The mean age was significantly higher (P<0.001) in older HCs (60.5±10.2 years) in comparison to younger HCs (22.9 ± 3.5 years). The indexed volumetric LV parameters were significantly reduced in old HCs, including indexed LV mass (77.6±14 g/m ^2 ^vs 116±19 g/m ^2^, P=0.005), LV end-diastolic volume (EDV) (146±40 ml/m ^2^ vs 194±43 ml/m ^2^, P=0.006), LV end-systolic volume (ESV) (55±16 ml/m ^2^ vs 82±29 ml/m ^2^, P=0.010), and LV stroke volume (SV) (84±22 ml/m ^2^ vs 104±35 ml/m ^2^, P=0.014). The indexed aortic forward flow was significantly reduced in old HCs than young HCs (38±15 ml/m ^2^ vs 48±14 ml/m ^2^, P=0.001). Similarly, significant differences were observed in FDs _avg_ (16±5% vs 8±4%, P<0.001), sFRR (7±6% vs 2±2%, P<0.001) and PWV (11±8 m/s vs 4±2 m/s, P<0.001) between the two groups ( Table 1 & Table 2).

**Figure 2.**
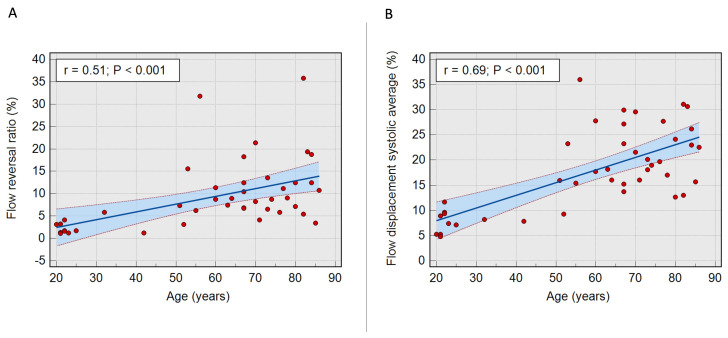
**A** - Scatter plot with 95% confidence interval illustrating a direct correlation between age and systolic flow reversal ratio (P<0.001). **B** - Scatter plot with 95% confidence interval illustrating a linear correlation between age and flow displacement systolic average (P<0.001).

Flow reversal ratio systolic average (sFRR), a marker of vorticity and reduced aortic conduit function, and FDs _avg_, a marker of flow eccentricity, were independently associated with age (P<0.001 and P<0.0001, respectively) ( Figure 2). sFRR was not significantly different between males and females (6.3±8.8% vs 8.7±5.3%, P=0.21). Similarly, there was no statistically significant difference in FDs _avg_ between males and females (15.5±12% vs 21.5±10%, P=0.066).

**Figure 3.**
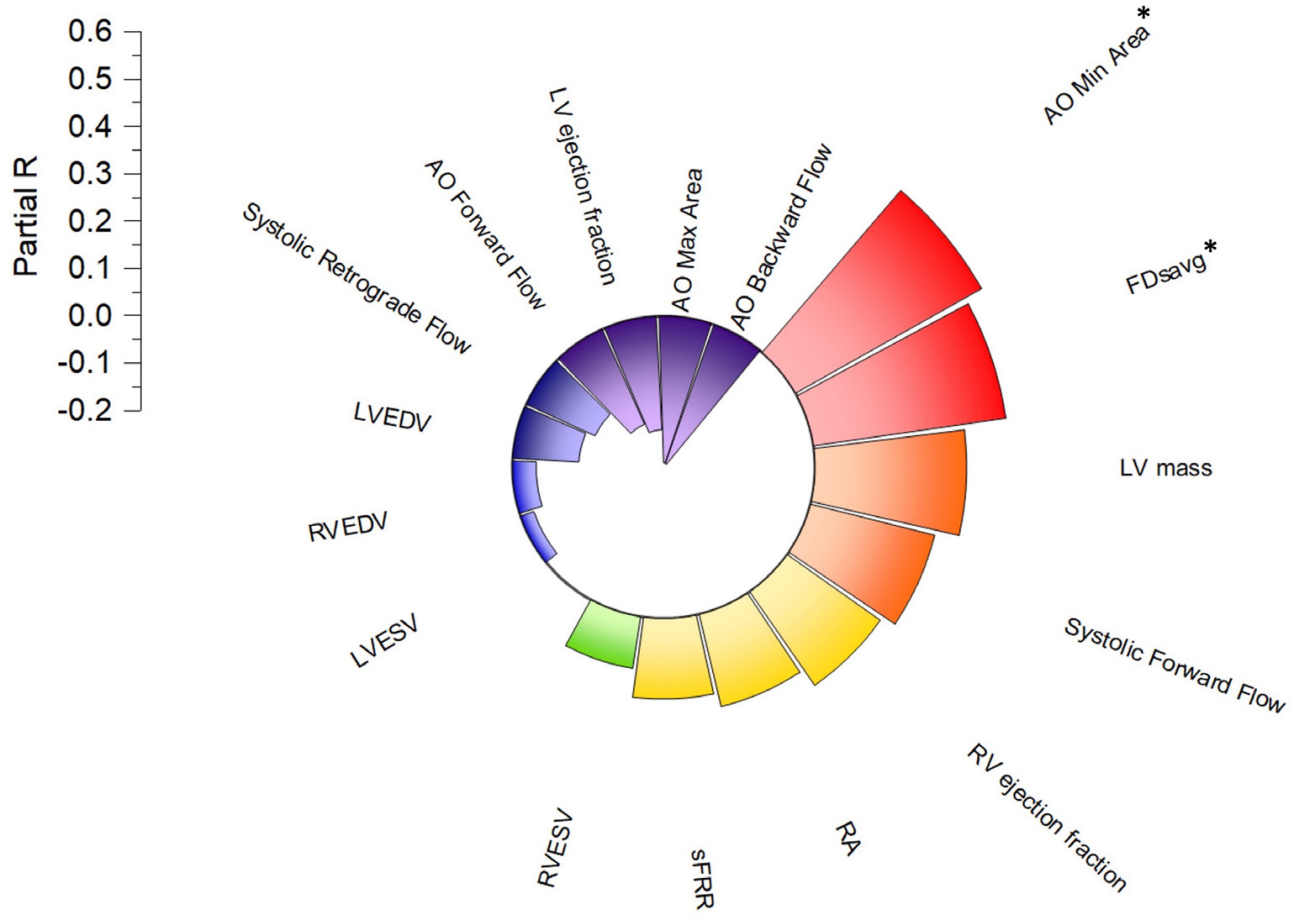
Radial bar plot illustrating a linear partial independent correlation of age with left ventricular and aortic flow parameters. Aortic minimum area (AO min area) and FDsavg have independent association to age of the whole study cohort and both increase with age.

On multiple regression analysis using Enter method aortic minimal area (partial R=0.45, P=0.01) and FDsavg (partial R=0.41, P=0.03) were the only two independent variables associated with age while factoring in all other CMR indices as covariates ( Figure 3).

### Aortic flow indices in Old HCs vs HFpEF

**Figure 4.**
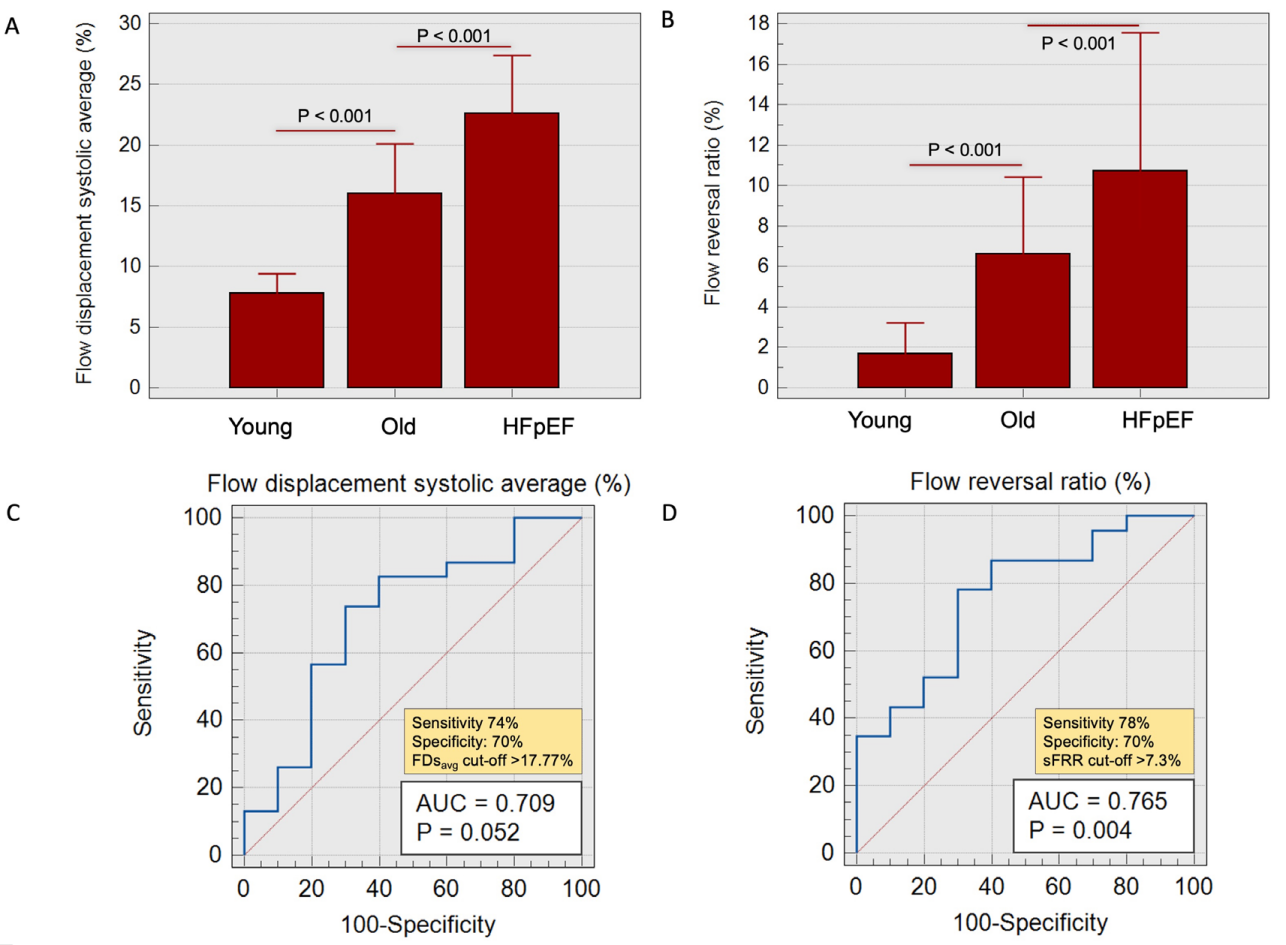
**A** – Bar charts demonstrating flow displacement systolic average trends in young vs old healthy cohorts vs HFpEF patients. **B** - Bar charts demonstrating systolic flow reversal ratio trends in young and old healthy cohorts and HFpEF patients. **C** and **D** - Receiver operating characteristic (ROC) with area under the curve demonstrating acceptable correlation in old HCs vs patients with HFpEF.

The FDs _avg _was significantly increased in HFpEF patients in comparison to old HCs (23±10% vs 16±5%, *P*<0.001) ( Figure 4). Additionally, sFRR was significantly elevated in HFpEF group vs old HCs (11±10% vs 7±6%, P<0.001). The RA was observed to be distinctly higher in HFpEF patients when compared to old HCs (16±48° vs -37±16°, P=0.04). However, there was no significant difference observed in the indexed aortic forward flow and PWV between these two groups.

A FDs _avg_ of >17.7% showed 74% sensitivity and 70% specificity in differentiating between old controls and patients with HFpEF (AUC=0.71, P=0.05). Similarly, a sFRR of >7.3% showed 78% sensitivity and 70% specificity in distinguishing between old controls and HFpEF (AUC=0.76, P=0.004).

### Aortic flow indices in Old HCs vs Age-gender-matched HFpEF

**Figure 5.**
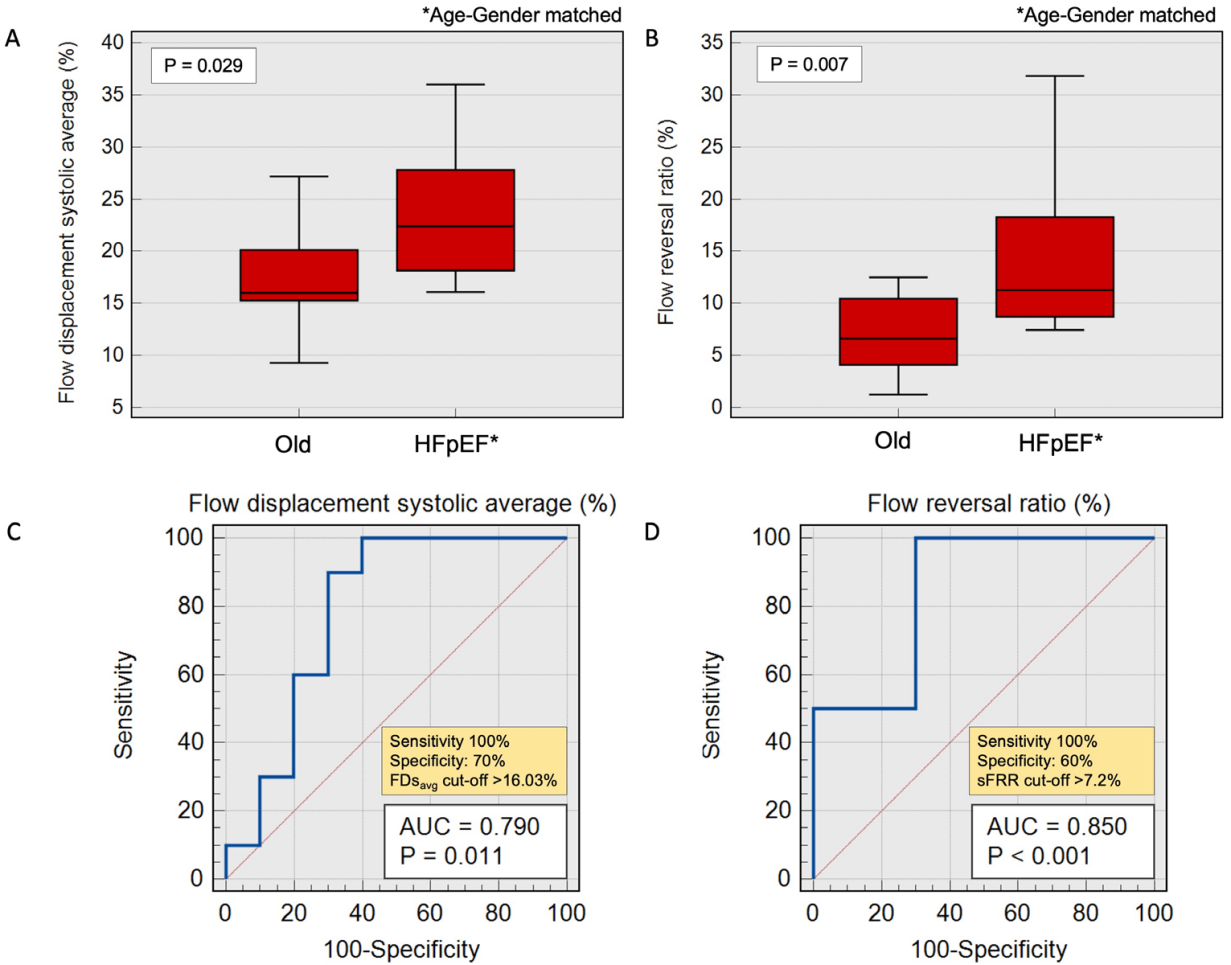
**A** – Bar charts demonstrating average systolic flow displacement trends in old healthy cohorts and age-gender-matched HFpEF patients. **B** - Bar charts demonstrating systolic flow reversal ratio trends in old healthy cohorts vs age-gender-matched HFpEF patients. **C** and **D** - Receiver operating characteristic (ROC) with area under the curve demonstrating acceptable correlation in old HCs vs patients with age-gender-matched HFpEF.

The differences in FDs _avg _and sFRR remained consistent and significant between HFpEF patients and old HCs when matched by age and gender. We observed a noticeable increase in FDs _avg_ in age-gender matched HFpEF patients (N=10) vs old HCs (22.4±9.7% vs 16±4.9%, *P*=0.029) ( Figure 5). Furthermore, sFRR was significantly elevated in age-gender-matched HFpEF patients vs old HCs (11.3±9.5% vs 6.6±6.4%, *P*=0.007). However, there was no significant difference observed in the indexed aortic forward flow, PWV and RA among these two groups. ( Table 3).

**Table 3.** Aortic flow indices in age-gender-matched old healthy cohort versus HFpEF.

	Old HCs N=10	HFpEF N=10	P-value
AO Forward Flow, ml	69.7±17.6	66.7±25.6	0.529
AO Forward Flow indexed, ml/m ^2^	38±15	35.1±11.4	0.529
AO Backward Flow, ml	1.1±3.6	2.1±2.8	0.436
AO Backward Flow indexed, ml	0.6±2.1	1±1.3	0.481
AO Max Area, mm ^2^	8.8±2.6	8.2±3.8	0.734
AO Min Area, mm ^2^	6.8±1.5	6.9±3.2	0.496
Flow Displacement systolic average, %	16±4.9	22.4±9.7	0.029 *
Rotational Angle, °	-3.3±16.2	4.7±39.5	0.579
Systolic Forward Flow, ml	66.4±18	66.7±25.3	0.529
Systolic Retrograde Flow, ml	4.2±4.4	8.3±5.7	0.017 *
Systolic Flow Reversal Ratio, %	6.6±6.4	11.3±9.5	0.007 *
Pulse Wave Velocity, m/s	11±8.1	8.3±0.1	0.727

Data were represented as median ± IQR (%). *AO* aorta, *HCs* healthy cohorts, *HFpEF* heart failure with preserved ejection fraction. *P<0.05 compared young HCs versus old HCs; ^#^P<0.05 compared old HCs versus HFpEF.

A FDs _avg_ exceeding 16% exhibited 100% sensitivity and 70% specificity in discerning age-gender matched HFpEF patients from old HCs (AUC=0.79, P=0.01). Correspondingly, a sFRR higher than 7.2% demonstrated a sensitivity of 100% and 60% specificity in distinguishing age-gender matched HFpEF patients from old HCs (AUC=0.85, P<0.001).

## Discussion

This study is one of the first to explore shifts in aortic flow hemodynamics within the ageing population and patients with HFpEF. A significant finding of this study is that both ageing and HFpEF demonstrate a rise in the systolic flow reversal ratio, which is a marker of aortic conduit function. This aortic flow biomarker, which also represents turbulent flow, exhibited marked disparities between old control group and age-gender-matched HFpEF patients. Moreover, FDs _avg_, an indicator of flow eccentricity in the ascending aortic root, increased progressively from young to old controls to HFpEF with marked differences between old controls and age-gender-matched HFpEF patients. Aortic forward flow also decreased in all respective groups despite no significant difference in left ventricular ejection fraction. We observed that the associations of sFRR and FDs _avg_ were aligned with ageing rather than LV functional parameters. These CMR-derived aortic flow biomarkers have high sensitivity to detect HFpEF early in the disease process and contribute to improved phenotyping.

### Mechanism underpinning Systolic Flow Displacement

Increased flow eccentricity in the aorta causes turbulence resulting in energy dissipation, requiring the heart to expend more energy to maintain sufficient blood flow making the cardiovascular system inefficient ^22, 23, 26, 27^. The clinical utility of FDs _avg_ as an independent biomarker has been established in predicting the rate of aortic growth in patients with bicuspid aortic valve (BAV) and aortopathy (with and without aortic valve disease) ^7, 27– 30^. We speculate that flow eccentricity, measured by FDs _avg_, develops due to left ventricular outflow tract remodelling and aortic remodelling due to an age-associated increase in afterload conditions on the ventricle ^31– 33^.

### Mechanism underpinning Systolic Flow Reversal

Elevated sFRR, a marker of reduced aortic conduit function and increased vorticity, has been independently linked to aortic root dilatation ^34^ and shows a linear relationship with the severity of aortic stenosis ^35^. Barker *et al.* ^35^ demonstrated an FRR of >10% in patients with BAV and significant aortic stenosis. The systolic flow reversal ratio measures the retrograde flow of vorticity and represents areas of low-pressure development near the inner curvature of the aortic root due to pressure equalisation, which will plausibly happen if left ventricular filling pressures are high or if, during systolic contraction, the left ventricle is not able to generate enough mechanical force ^36^, for example, after myocardial infarction. These underlying pathophysiological processes have been described in previous computational fluid dynamics simulation studies ^37^. In our HFpEF cohort, 48% of patients had systemic hypertension, and 35% had a previous myocardial infarction. 

Our study is the first to show that abnormal aortic hemodynamics correlates with age and HFpEF without significant aortic valve disease. We observed that an sFRR level greater than 7.3% was found to have a sensitivity of 78% and specificity of 70% in distinguishing between old controls and HFpEF. Moreover, an FDs _avg_ level greater than 18% had a sensitivity of 74% and specificity of 70% for the same differentiation. This study highlights that the mechanism of both flow eccentricity and flow turbulence is not only associated with the stenotic aortic valve. Interestingly, this study did not reveal any significant difference in PWV between old controls and HFpEF, contrary to previous studies ^38, 39^.

### Ageing signatures of CMR

Recently there has been a lot of interest in ageing-associated CMR signatures as ageing in itself is associated with cardiovascular outcomes. In a recent study by Shah, M. *et al.* (2023), machine learning was employed to forecast biological age by analysing multiple cardiovascular characteristics from CMR images and electrical data in 39,599 participants ^40^. The study found that aging was associated with left ventricular and aortic stiffness, both of which emerged as a robust predictor of deviation from healthy cardiovascular aging and associated with range of cardiovascular outcomes. In addition, more recently, in a cohort of 169 healthy individuals, Zhao et al have demonstrated how both FDsavg and sFRR are associated with exercise capacity assessed by peak oxygen uptake (PVO2) from cardiopulmonary exercise testing (r=-0.302, r=-0.219 PP<0.05) ^41^. With increase in both this aortic flow abnormalities, PVO2 decreases. The findings from their research, along with our study, support the notion that breathlessness in patients with HFpEF is correlated with aortic flow abnormalities.With the growing literature, FD and sFRR analysis can be applied to 2D phase-contrast imaging, extending their applicability to wider and more diverse populations to define this causation better.

### Clinical impact

Current echocardiogram models to estimate left ventricular filling pressure remain complex and factor in right heart haemodynamics, including tricuspid regurgitation velocity, which will get affected in the end-stage of HFpEF. An increase in right-sided pressure will result after the whole pulmonary vascular bed has been adversely remodelled, leading to a late diagnosis of HFpEF. The utility of 4D Flow CMR to quantify aortic flow hemodynamics (FDs _avg_ and sFRR) could aid in the early diagnosis of HFpEF and sub-phenotyping patients with HFpEF. This is important as it will allow early pharmacological intervention and improve clinical outcomes. Moreover, FDs _avg _and FRR can be easily applied to 2D phase contrast CMR, making them widely applicable for more extensive studies. Future studies are required to evaluate the direct link between aortic flow physiology and exercise capacity. We must also establish how aortic flow influences left ventricular filling and central aortic pressures.

## Limitations

While our observational and case-controlled study suggests that there may be a link between aortic flow abnormalities with ageing and HFpEF, it is important to note that the sample size used in this study was relatively small, which limits the generalisability and calls for further research with larger and more diverse cohort to draw definitive conclusions. There is a possibility of selection bias in the HFpEF cohort, and we may be identifying individuals who are much further down the temporal changes of ageing associated with HFpEF. Nevertheless, by doing age-matched comparison, we still are able to demonstrate patho-physiological step-up changes in different ages and diseased state. With no intervention currently available to improve aortic flow hemodynamics, our study is limited in establishing causality and restricts observations to only correlations. It is possible that other prevalent systemic risk factors in HFpEF, such as hypertension, obstructive sleep apnoea (OSA), diabetes, and obesity, could also contribute to changes in flow displacement and sFRR. Therefore, caution must be exercised when interpreting these findings as they may not represent the boarder population with HFpEF and age-related changes in the cardiovascular system.

## Conclusion

Aortic flow haemodynamics, namely FDs _avg_ and sFRR, are significantly affected in ageing and patients with HFpEF. Incorporating aortic flow haemodynamics assessment in routine clinical practice may allow early and improved detection of HFpEF.

## Ethics approval and consent to participate

This study was conducted according to the principles outlined in the Declaration of Helsinki - Version 2013. The collection and management of data were approved by the National Research Ethics Service in the United Kingdom (21/NE/0149).

## Consent

All healthy controls gave informed consent. All patients were provided with clear information about the study. A pragmatic opt-out informed consent was obtained from all patients included in the study ^19^.

## Data Availability

The datasets generated and analysed during the current study are not publicly available. Access to the raw images of patients is not permitted since specialised post-processing imaging-based solutions can identify the study patients in the future. Data are available from the corresponding author upon reasonable request.
