# Supplementary material for: Aortic flow is abnormal in HFpEF
Source: Wellcome Open Res. 2024 Mar 6;8:577. Originally published 2023 Dec 15. [Version 2] doi: 10.12688/wellcomeopenres.20192.2 (PMC10940846; doi:10.12688/wellcomeopenres.20192.2)
Supplement: Peer review history

# Reviewer report: Reviewer response for version 2

**DOI:** 10.21956/wellcomeopenres.23435.r117787

**Published:** 2025-02-13

**Authors:** João Filipe Fernandes

**Affiliations:** 1 King's College London, London, UK

The work presented Mehmood et al is very interesting and unique, as for the first time, to my knowledge, it relates changes in aortic flow due to Heart Failure with preserve ejection fraction (HFpEF). The article is clearly written and well-illustrated. In a group of 43 subjects (with 20 being age-matched controls, and 23 being HFpEF) undergoing CMR with 4D Flow MRI acquisition, the pulse wave velocity (PWV) and blood flow profiles at the root of the aorta artery, via aortic flow displacement (FDsavg) and systolic flow reversal ratio(sFRR) artery were studied. There was significant differences found between controls and HFpEF patients for all metrics with the small nuance that for age-matched controls vs HFpEF there was no difference in PWV. The Methodology can be implemented in standard 4D Flow MRI acquisitions, which might open the door for studies with higher number of patients and further cardiac pathologies or HFpEF sub-phenotyping, as well as potential widespread use in clinical routine in the longer term, which might enable earlier detection of HFpEF. In my view, and after addressing the following comments satisfactorily, I believe this work should be indexed, specifically considering the smart use of key arterial flow markers to a condition that is still misunderstood, as is the case of HFpEF.

 There are a couple of comments that I would like to make, and that can potentially increase further the quality of the study without extensive re-analysis.

  

- Since the minimum and maximal areas were obtained, I would recommend computing the compliance and distensibility of the aortic root to complement the PWV (please find the link under for the paper by Ghorbani et al), if aortic pressure (pulse pressure in specific) was also obtained via brachial Riva-Rocci (or identical) before or after the MRI acquisition.
- As a previous reviewer noted before, these results are not quantifications of turbulent flow, as turbulent flow is undetectable in clinically available 4D Flow MRI sequences. However, there is initial in-vitro and in-silico evidence, in a paper by Fernandes et al (link under), that momentum variation is a surrogate of turbulent dissipation, especially in a region where there is pressure recovery (as the root of the aorta after vena contracta), the change in momentum is proportional to the turbulent dissipation. By other words, while kinetic energy (velocity) is being reduced over space after vena contracta (where velocity is maximal), there is an energy transfer into potential energy (pressure recovery) and into viscous and turbulent dissipation. As the momentum quantification depends on the cross-sectional blood flow profile, which in turn is affected by Flow Displacement and Systolic Retrograde Flow, it is then logic that the biomarkers presented in the paper are indeed associated with turbulent dissipation in the aorta.
- There were ROIs contouring a cross-section of the aortic arch and the descending aorta (to compute PWV). Did the authors did the same analysis in these ROIs. If yes, the results could be brought into an appendix and mention it briefly in the discussion.
- Authors can comment if LV was part of the FOV of the 4D Flow MRI, and if yes, if the quantification of the Kinetic Energy could additionally be used to characterize HFpEF patients undergoing 4D Flow MRI acquisition.
- The fact that the younger control group is unbalance from a gender perspective should be part of the limitations.
- In Figure 1, panels a and c, an anatomical reference should be given to understand better the cross-section view (for example annotating the anterior and right-left directions with letter). This is specially interesting to understand where is the AV located in relation to eccentric flow of panel c. Also indicate if the heat-maps come from a specific time of the cycle or if are an average of the systolic flow profile.
- In Figure 2, it could be interesting to colour each data point based on the subject belonging to the control groups or the HFpEF groups. Not sure if having 2 correlations could help to see different trends, but such would compromise the rapid understanding of the figure.

Also note, that due to the data being inherently identifiable even if images are anonymized, its open publication is not possible. The authors explain this aspect well.

Is the work clearly and accurately presented and does it cite the current literature?

Yes

If applicable, is the statistical analysis and its interpretation appropriate?

Yes

Are all the source data underlying the results available to ensure full reproducibility?

Partly

Is the study design appropriate and is the work technically sound?

Yes

Are the conclusions drawn adequately supported by the results?

Yes

Are sufficient details of methods and analysis provided to allow replication by others?

Yes

Reviewer Expertise:

4D Flow MRI, Biomedical Engineering, Heart Failure, CMR, echocardiography.

I confirm that I have read this submission and believe that I have an appropriate level of expertise to confirm that it is of an acceptable scientific standard.

## References

[1] : Impact of valve morphology, hypertension and age on aortic wall properties in patients with coarctation: a two-centre cross-sectional study. *BMJ Open* .2020;10(3) : 10.1136/bmjopen-2019-034853 e034853 10.1136/bmjopen-2019-034853 32213521 PMC7170596

[2] : Non-invasive cardiovascular magnetic resonance assessment of pressure recovery distance after aortic valve stenosis. *J Cardiovasc Magn Reson* .2023;25(1) : 10.1186/s12968-023-00914-3 5 10.1186/s12968-023-00914-3 36717885 PMC9885657

# Reviewer report: Reviewer response for version 2

**DOI:** 10.21956/wellcomeopenres.23435.r115328

**Published:** 2025-01-22

**Authors:** Jan Engvall

**Affiliations:** 1 Linköping University, Linköping, Sweden

I thank the editors for the opportunity to assess this manuscript which has received approval by 2 previous reviewers. The manuscript deals with aortic flow patterns in heart failure with preserved ejection fraction.

***Title and Abstract***

 Title is OK. Abstract could contain AUC for the detection of HFpEF. It is easier to understand a combination of sensitivity and specificity than to grasp AUC, but then it is more common to report sensitivity at a specificity of 80% instead of reporting sensitivity at a specificity of 70%.

***Methods***

 Patients in this study have been sourced from the PREFER-CMR registry in Norfolk and Norwich University Hospitals, initiated in 2022. The trial is registered in ClinicalTrials, but that entry has not been followed up. From the home page, it seems that 800 patients are included in the PREFER-CMR registry. All patients undergoing CMR at this institution are invited to participate in the registry. The standard CMR-study includes 4D-flow sequences, whenever possible. From the registry, various research questions can be adressed based on use of previously collected patient images and clinical data. In this particular study, 20 healthy controls (10 young=23 y.o., 10 old=61 y.o.) were selected from the registry in addition to 23 patients with a diagnosis of HFpEF (mean age 74 y.o.). A diagnosis of HFpEF was based on the presence of signs and symptoms of heart failure and certain CMR criteria previously developed by this group (fulfilling a regression equation that involves LA volume and LV mass indicating the probability of having LVFP >15 mm Hg).

 A better description of the diagnosis in the HFpEF patients has been asked for by a previous reviewer. However, the need for multiple inputs (echocardiographic findings, NT-pro-BNP levels), the difficulty in the diagnosis of HFpEF and the presence of diagnoses mimicking HFpEF has not been pursued by the authors in this text. Since the comparison of differences in 4D flow is highly dependent on the selection of the HFpEF group, the reader needs a more detailed description of patient selection.

 The CMR method is well described but the authors fail to cite the features of quality control that are recommended in the 2023 update of guidelines on CMR 4D-flow by Bissel et al, summarized in Table 3 of that publication (dr Garg is co-author). I lack e.g. a number on the reconstructed voxel size (acquired is 3x3x3 mm).

 Systolic flow reversal in the ascending aorta is illustrated in Figure 1 but it could benefit the reader if anatomical directions were added to part a and c of the figure, e.g. anterior-posterior, lateral-septal. What is the meaning of flow reversal at peak systole in Figure 1? Is it part of excessive vortex development that could perhaps be related to a less pronounced Windkessel effect in older people, due to increased wall stiffness or reflected pressure waves? Could you try to illustrate vortex flow e.g. by a pathline analysis in the ascending aorta?

***Results***

 A number of flow measures are given in Table 1 and Table 2. A comparison is performed between young and old healthy controls and with the HFpEF group. For young healthy compared to old/oldHFpEF, stroke volume is higher whether measured anatomically or from 4D/2D flow which is expected. Flow reversal is according to Table 1 and 2 rather small, but increases with age. In Table 2, the aortic area max/min is shown but the figures are very low 7/5 mm2 for young healthy which corresponds to 2.5x2.5 mm diameter, which needs an explanation.

***Discussion***

 Under the subheading “Mechanism underpinning Systolic Flow Reversal” the authors discuss pressure equalization and remodelling of the LVOT as an explanation for flow reversal, but do not discuss vorticity in relation to other features such as reflected pressure waves.

 Under the subheading “Ageing signatures of CMR” the authors suggest that “breathlessness in patients with HFpEF is correlated  with  aortic  flow  abnormalities”. The association might be true, but the mechanism causing breathlessness has to be sought elsewhere, e.g. in the stretch receptors of the pulmonary circulation.

 In summary, this work is interesting but some questions remain as to the selection of the HFpEF patients and the quality control performed on the 4D-acquisition. There are also some questions as to aortic area in Table 2. I believe that citing the guideline paper of Bissel certifying that recommendations regarding quality control of scientific studies in CMR 4D-flow have been followed would strengthen the impact of the text.

Is the work clearly and accurately presented and does it cite the current literature?

Partly

If applicable, is the statistical analysis and its interpretation appropriate?

Yes

Are all the source data underlying the results available to ensure full reproducibility?

Yes

Is the study design appropriate and is the work technically sound?

Yes

Are the conclusions drawn adequately supported by the results?

Yes

Are sufficient details of methods and analysis provided to allow replication by others?

Yes

Reviewer Expertise:

CMR ventricular function, CMR 4D-flow, Stress echocardiography, Transthoracic and transesophageal echocardiography, Epidemiology

I confirm that I have read this submission and believe that I have an appropriate level of expertise to confirm that it is of an acceptable scientific standard, however I have significant reservations, as outlined above.

## References

[1] : 4D Flow cardiovascular magnetic resonance consensus statement: 2023 update. *J Cardiovasc Magn Reson* .2023;25(1) : 10.1186/s12968-023-00942-z 40 10.1186/s12968-023-00942-z 37474977 PMC10357639

# Reviewer report: Reviewer response for version 2

**DOI:** 10.21956/wellcomeopenres.23435.r116687

**Published:** 2025-01-15

**Authors:** Sungho Park

**Affiliations:** 1 University of Colorado Anschutz Medical Campus, Aurora, Colorado, USA

The authors have demonstrated the impact of aging and heart failure with preserved ejection fraction (HFpEF) on left ventricular (LV) function and aortic hemodynamics using cardiac MRI, 2D phase-contrast (PC), and 4D flow MRI techniques. This study introduces flow displacement and flow reversal ratio as novel biomarkers for understanding aging and HFpEF, which is highly interesting. However, several concerns need to be addressed before indexing.

 First, turbulent flow is characterized by unsteady fluid motion and chaotic behavior. While swirling and retrograde flow can be features of turbulent flow, they are not direct markers as they may also appear in laminar flow. If the participants, including old healthy controls and individuals with HFpEF, demonstrate high peak velocities, this could support the presence of turbulent flow, which could be confirmed by calculating the Reynolds number. However, given that these participants do not appear to have valvular disease and show a decreasing trend in stroke volume (as indicated in Table 1), peak velocity might not be high enough to classify the aortic flow as turbulent. The authors should clarify the presence or absence of aortic valve disease, revise Figure 1c,d to better represent the data (as the current figures include an extreme forward flow case not consistent with the averages in Table 1), and verify the values or units in Table 1, as some markers seem not to be indexed. Based on these considerations, terms like "turbulent flow" and "energy dissipation" might not be appropriate in the introduction and discussion sections, and alternative mechanisms or terminologies should be proposed.

 Additionally, the authors mentioned that 2D PC MRI data was used when 4D flow data was unavailable, but the manuscript lacks sufficient details on this. It is important to specify how many 2D PC MRI datasets were used in each group and whether the sample sizes were adequate to compare results between 4D flow and 2D PC MRI. The sequence parameters for 2D PC MRI should also be detailed.

 The study reported no significant differences in pulse wave velocity (PWV) between old healthy controls and individuals with HFpEF, highlighting discrepancies with previous studies. However, as the current study measured aortic PWV while previous studies often measured carotid-to-femoral PWV, it is plausible that aortic stiffening may not manifest in HFpEF, while carotid-to-femoral vasculature shows stiffening. The authors should clarify this distinction and provide insights into the underlying mechanisms behind these observations.

 Numerous typographical errors remain in the manuscript and should be carefully addressed.

Is the work clearly and accurately presented and does it cite the current literature?

Partly

If applicable, is the statistical analysis and its interpretation appropriate?

Partly

Are all the source data underlying the results available to ensure full reproducibility?

Partly

Is the study design appropriate and is the work technically sound?

Partly

Are the conclusions drawn adequately supported by the results?

Partly

Are sufficient details of methods and analysis provided to allow replication by others?

Partly

Reviewer Expertise:

Biomedical engineering; 4D flow MRI; MRI imaging

I confirm that I have read this submission and believe that I have an appropriate level of expertise to state that I do not consider it to be of an acceptable scientific standard, for reasons outlined above.

# Reviewer report: Reviewer response for version 2

**DOI:** 10.21956/wellcomeopenres.23435.r76057

**Published:** 2024-03-14

**Authors:** Jonathan Bennett

**Affiliations:** 1 University College London, London, UK

The authors have clarified the selection criteria for HFpEF patients combining symptomatic status and recently developed CMR estimations of pulmonary capillary wedge pressure. Additionally, data has been provided comparing 2D and 4D measurements with a low in-subject variation and co-efficient of the mean.

Is the work clearly and accurately presented and does it cite the current literature?

Yes

If applicable, is the statistical analysis and its interpretation appropriate?

Yes

Are all the source data underlying the results available to ensure full reproducibility?

No

Is the study design appropriate and is the work technically sound?

Yes

Are the conclusions drawn adequately supported by the results?

Yes

Are sufficient details of methods and analysis provided to allow replication by others?

Yes

Reviewer Expertise:

Cardiac magnetic resonance, aortic valve diesease, HFpEF, aging, preventive cardiology, heart-brain interactions.

I confirm that I have read this submission and believe that I have an appropriate level of expertise to confirm that it is of an acceptable scientific standard.

# Reviewer report: Reviewer response for version 1

**DOI:** 10.21956/wellcomeopenres.22355.r72474

**Published:** 2024-03-01

**Authors:** Jonathan Bennett

**Affiliations:** 1 University College London, London, UK

This article investigates age-related and heart failure with preserved ejection fraction (HFpEF) related differences in aortic flow dynamics. It aims to describe these variances by assessing novel markers of aortic flow hemodynamics using cardiac magnetic resonance (CMR) two-dimensional phase contrast flow measurements in young healthy controls, older healthy controls, and HFpEF patients.

*Title and abstract:*

 The title is short and non-descriptive of the methods or type of abnormal flows detected – the authors could expand the title to give potential readers more insight into the article.

 The abstract is clear and succinct and relates to the findings reported in the full manuscript.

 The authors state the use of two-dimensional phase contrast CMR data as the method of acquisition in the abstract, however methods state that the data was derived from an initial 4D flow acquisition – the authors could be more specific in this respect.

*Introduction:*

 The introduction is clear with its rationale for the study, having identified that aortic flow hemodynamics are abnormal in bicuspid aortic valve disease and that this relates to impaired aortic conduit function. The main objective for the study is clearly stated.

*Methods:*

 The HFpEF cases were included based upon a confirmed diagnosis of HFpEF by CMR – the authors could expand on what would be considered diagnostic of HFpEF on CMR considering it is less commonly used compared to echocardiography. Also, an explicit statement of the symptomatic status of HFpEF patients for clarity.

 The CMR protocol and analysis is well described. The authors expertly describe the various aortic hemodynamic parameters measured in bullet point form that is very useful for the reader.

 The authors state that if 4D flow wasn’t available 2D phase-contrast flow was used instead, some clarity regarding whether some patients had 2D phase-contrast compared with the 2D phase-contrast derived from 4D acquisition would be beneficial, and whether there was any analysis as to whether impact on results.

 Figure 1 is clear in illustrating the methods.

*Results:*

 The HFpEF group is reported as having 12 (52%) atrial fibrillations, and presumably the other groups all in sinus rhythm. It is not explicit as to what rhythm the HFpEF patients were in during image acquisition and the effect that this may have on the aortic flow haemodynamics – a statement on how atrial fibrillation was dealt with within the study would be useful.

 The results section is clearly and logically presented taking the reader through the results of the main groups. The authors do further sub-group analysis of age-matching to confirm that aortic haemodynamic differences between these groups are unlikely to be a factor of age.

 Figures are clear and well described.

*Discussion:*

 The authors state that the aortic haemodynamics have a higher sensitivitiy to detect changes early in the HFpEF disease process – however it is unclear as to how they came to this conclusion and what they would define as early HFpEF and whether this is how they would define their HFpEF cohort.

 The limitations are well described and highlight the small sample sizes used in the study that limits the generalizability in the study, however considering the relatively novel nature of the measurements in these populations this study serves as a good starting point for future research.

*Ethics:*

 No ethical issues noted.

*Summary:*

 The authors present a well-described and analyzed comparison of aortic flow hemodynamics in three distinct cohorts, identifying potential future biomarkers. The methodology is robust, and writing style is clear, guiding the reader through the manuscript. They acknowledge limitations in sample size and address age differences between the cohorts. 

 Areas for clarification:

  

- Specific inclusion criteria for HFpEF via CMR, an uncommon technique for identifying HFpEF.
- Integration of atrial fibrillation within the HFpEF cohort into aortic flow measurements and its potential impact on results.
- Definition of "early HFpEF" and its relevance to the HFpEF group in this study.
- Clarification on whether all patients underwent 4D flow acquisition converted to 2D phase contrast or if some only had initial 2D phase contrast acquisition, and any analysis conducted to assess methodological differences.

Addressing these points will enhance the paper's value by providing early insights into potential biomarkers and mechanisms in HFpEF for future research in this critical field.

Is the work clearly and accurately presented and does it cite the current literature?

Yes

If applicable, is the statistical analysis and its interpretation appropriate?

Yes

Are all the source data underlying the results available to ensure full reproducibility?

No

Is the study design appropriate and is the work technically sound?

Yes

Are the conclusions drawn adequately supported by the results?

Yes

Are sufficient details of methods and analysis provided to allow replication by others?

Yes

Reviewer Expertise:

Cardiac magnetic resonance, aortic valve diesease, HFpEF, aging, preventive cardiology, heart-brain interactions.

I confirm that I have read this submission and believe that I have an appropriate level of expertise to confirm that it is of an acceptable scientific standard, however I have significant reservations, as outlined above.

# Response

**Published:** 2024-03-02

**Authors:** Pankaj Garg

**Affiliations:** Norwich Medical School, University of East Anglia, Norwich, England, UK

Areas for clarification: The title is short and non-descriptive of the methods or type of abnormal flows detected – the authors could expand the title to give potential readers more insight into the article. **Author's Response: **We appreciate the reviewer’s feedback regarding the brevity of the title. It is important to recognise that a concise title can serve as an effective attention-grabber, enticing readers to explore the content further. While the title provides an initial touchpoint, the article itself delves into comprehensive discussions on the methods employed and the various types of abnormal flows detected. By maintaining a succinct title, we encourage readers to engage with the full text, ensuring a deeper understanding beyond what an extended title might convey. Specific inclusion criteria for HFpEF via CMR, an uncommon technique for identifying HFpEF. **Author's Response: **We appreciate the reviewer’s feedback regarding the initial manuscript. In response, we have provided additional clarity on the definition of Heart Failure with Preserved Ejection Fraction (HFpEF). Specifically, we now elaborate on the diagnostic criteria, which include a confirmed clinical diagnosis based on symptoms, signs, and Cardiovascular Magnetic Resonance (CMR) features associated with HFpEF (mainly raised left ventricular filling pressure >15mmHg). Notably, CMR features such as increased left atrial size and left ventricular hypertrophy serve as hallmarks of HFpEF. Importantly, the utilisation of a composite equation for left ventricular filling pressure through CMR enables more accurate assessments compared to traditional echocardiography indices) [PMID: 35512290]. Consequently, we have opted for CMR as the preferred diagnostic modality. We are sorry that the initial manuscript did not clarify this in detail. Integration of atrial fibrillation within the HFpEF cohort into aortic flow measurements and its potential impact on results. **Author's Response: **We appreciate the reviewer’s attention to the details of our study. In response, we have provided further clarification regarding the inclusion criteria for patients with atrial fibrillation (AF). Specifically, we ensured that our study cohort consisted exclusively of AF patients with stable heart rates and good image quality. Patients with significant R-R variability were deliberately excluded to maintain consistency in our data. Regarding the impact of R-R variability, we acknowledge its potential to introduce temporal blurring during image acquisition, particularly during diastolic phases. However, we emphasise that the majority of the parameters proposed in our study are unlikely to be significantly affected by AF-related variability. Notably, the systolic phases remain less susceptible to this issue. Nevertheless, it is essential to recognise that the independent role of AF-associated hemodynamics and its specific impact on aortic flow indices warrant further investigation. Regrettably, our study remains underpowered to evaluate this aspect fully. We appreciate the reviewer’s consideration of this limitation and will continue to explore this area in future research endeavours. Definition of "early HFpEF" and its relevance to the HFpEF group in this study. **Author's Response: **We appreciate the thoughtful consideration of our study. The observed continuum in our cohort underscores an essential aspect: the older group studied is highly likely to develop symptoms associated with Heart Failure with Preserved Ejection Fraction (HFpEF) at some point. Our interpretation suggests that patients with the HFpEF syndrome experience an accelerated aging process within the cardiovascular system. This process manifests as reduced ventricular and aortic compliance, leading to subtle aortic flow abnormalities. Importantly, these abnormalities may already be evolving in older subjects who have not yet reached the critical symptomatic threshold. While intriguing, we acknowledge that our study does not establish a direct link between aortic flow abnormalities and associated comorbidities. Such an investigation remains beyond the scope of our current work. However, we recognise the potential clinical implications. The methods proposed in our study may serve as early indicators, hinting at the development of HFpEF. Future research endeavours should explore this link comprehensively, bridging the gap between aortic flow dynamics and clinical outcomes. Clarification on whether all patients underwent 4D flow acquisition converted to 2D phase contrast or if some only had initial 2D phase contrast acquisition, and any analysis conducted to assess methodological differences. **Author's Response: **We indeed carried out internal validation to check if 4D flow re-formatted plane flows at a level similar to 2D phase-contrast through-plane flows give similar results. In a randomly selected cohort of five cases where both data were available, we noted good agreement between 4D flow and 2D PC methods for FDs _avg_ and sFRR. This has now been added to the results section of the manuscript. This cross-validation allowed in some cases of HFpEF, where 4D flow was not available, for us to use the 2D PC.

# Reviewer report: Reviewer response for version 1

**DOI:** 10.21956/wellcomeopenres.22355.r72475

**Published:** 2024-02-27

**Authors:** Nadir Elamin

**Affiliations:** 1 Sheffield Teaching Hospital, Sheffield, UK

This study is exceptional in its structure and presentation. The authors effectively introduced the topic of heart failure with preserved ejection fraction (HFpEF) and the significance of aortic flow. The hypotheses and objectives of the study were clearly outlined, making it easy to understand the purpose of the research. To enhance the introduction further, it would be beneficial to elaborate slightly on the methods used to evaluate aortic flow and provide illustrations of why echocardiography may not be suitable for intricate flow assessment.

 The methodology section is well-organised and clearly defined, allowing for seamless navigation between sections. The authors provided a comprehensive description of the study cohort and the standardised cardiovascular magnetic resonance (CMR) protocol, including image acquisition and data analysis. The different types of aortic flow assessments were also clearly defined in the methods section, and the statistical methods were well described, contributing to the reproducibility of the study.

 The presentation of the results was clear and concise, with appropriate tables and figures used to support the findings. Nevertheless, incorporating a video comparing the aortic flow in the three groups would be beneficial. The authors effectively interpreted the results and integrated them with existing literature, highlighting the significance of aortic flow in HFpEF patients and older healthy control cohort. This advances our knowledge in both areas and provides opportunities for future research.

 Overall, this study is of the highest quality, as evidenced by its coherent and well-organised structure. The paragraphs effectively summarised the objectives, and it is easy to create a list of bullet points from them. There are no grammatical or language errors that could impact the quality of the paper. This study will make a significant contribution to the literature on HFpEF patients, the innovative technology of CMR, and the importance of aortic flow in the aging population and patients with cardiovascular disease.

Is the work clearly and accurately presented and does it cite the current literature?

Yes

If applicable, is the statistical analysis and its interpretation appropriate?

Yes

Are all the source data underlying the results available to ensure full reproducibility?

Yes

Is the study design appropriate and is the work technically sound?

Yes

Are the conclusions drawn adequately supported by the results?

Yes

Are sufficient details of methods and analysis provided to allow replication by others?

Yes

Reviewer Expertise:

Anti-thrombotic therapy, Cardiovascular disease, Percutaneous coronary and structural intervention, Heart failure, Hamodynamic of the heart and vascular system.

I confirm that I have read this submission and believe that I have an appropriate level of expertise to confirm that it is of an acceptable scientific standard.
